# mSphere of Influence: The perfect slice—from pizza to proteases

**DOI:** 10.1128/msphere.00556-25

**Published:** 2025-10-09

**Authors:** Nicholas J. Lennemann

**Affiliations:** 1 Department of Microbiology, The University of Alabama at Birmingham 9968, Birmingham, Alabama, USA; Virginia-Maryland College of Veterinary Medicine, Blacksburg, Virginia, USA

**Keywords:** plus-strand RNA virus, orthoflavivirus, protease

## Abstract

Nick Lennemann studies the intracellular interactions of viral and host proteins. In this mSphere of Influence article, he discusses his employment history and the mentors that promoted his training and transition to an independent research program focused on proteolytic determinants of virus infection. He highlights how “A novel interaction between dengue virus nonstructural protein 1 and the NS4A-2K-4B precursor is required for viral RNA replication but not for formation of the membranous replication organelle” by A. Płaszczyca, P. Scaturro, C. J. Neufeldt, M. Cortese, et al. (PLoS Pathog 15:e1007736, 2019, https://doi.org/10.1371/journal.ppat.1007736) and “Species-specific disruption of STING-dependent antiviral cellular defenses by the Zika virus NS2B3 protease” by Q. Ding, J. M. Gaska, F. Douam, L. Wei, et al. (Proc Natl Acad Sci USA 115: E6310–E6318, 2018, https://doi.org/10.1073/pnas.1803406115) demonstrate the importance of viral protease activity for the establishment of a productive intracellular environment for infection.

## COMMENTARY

Making pizza was not my strong suit, and it still is not. However, in high school, several friends spent their evenings working at a local pizza parlor, so I figured it would be worth applying in order to work with my friends. While prepping, baking, and slicing up pizza was not my favorite job (definitely was not the worst), I did learn how important an active, collegial, and energetic environment is to being happy and productive. Like many colleagues in academia, I have worked numerous unfulfilling jobs in service, retail, physical labor, and finance—I was elated to discover that a career in molecular biology research was possible. This was only apparent after being fortunate to work with supportive mentors throughout my career.

Late in my undergraduate studies at Saint Cloud State University, I began performing research in a cell biology laboratory and was encouraged by my mentor, Dr. Brian Olson, to pursue graduate research in my academic passion—molecular virology. This led me to the University of Iowa to perform my doctoral research with Dr. Wendy Maury, where I studied the biochemical properties of filovirus glycoproteins. It was during this time that I became intrigued by the influence of host proteases on virus entry (e.g., cathepsins). This interest led me to seek a postdoctoral opportunity with Dr. Carolyn Coyne at the University of Pittsburgh, an expert in proteolytic regulation of enterovirus infection. During my interview with Dr. Coyne, she showed me transmission electron micrographs of coxsackievirus B and dengue virus-infected cells. These images immediately evoked questions of how individual viral proteins, liberated from large polyproteins, can result in the formation of extravagant membranous structures in the cell. These structures represent a hallmark of all positive-strand RNA virus infections, where they serve as a platform to concentrate host and viral proteins/factors required for replication and have been proposed to shield viral replication intermediates from cytoplasmic innate immune sensors. The mechanisms of the biogenesis of these *de novo* organelles continue to be an interesting avenue of ongoing research. After joining the Coyne laboratory, I was introduced to new techniques and research questions. Dr. Coyne encouraged me to pursue research opportunities that would promote a strong independent program merging my graduate training in molecular virology with new concepts and techniques in cell biology. During this time, there were two publications that influenced my research, which combined the techniques and interests I developed throughout my training from undergraduate to postdoctoral research.

In 2019, Płaszczyca et al. identified an essential intermediate of the orthoflavivirus polyprotein for viral replication (1). This study utilized innovative reverse genetics to perform a forward genetic screen that identified a novel viral polyprotein intermediate required for infection. The mechanistic experiments of this study identified that the interaction between the secreted nonstructural protein 1 (NS1) interacts in the lumenal space of the endoplasmic reticulum (ER) with a multi-transmembrane proteolytic intermediate of the polyprotein (NS4A-2K-4B). This study used molecular virology and microscopy techniques to show that the interactions between NS1 and the NS4A-2K-4B precursor, not the individual proteolytic products, are required for viral genome replication, but not the formation of the “peas in a pod” membranous replication structures, which are indicative of DENV infection. Their data piqued my interest in the complexities of viral polyproteins and the role of viral proteases in the regulation of replication and manipulation of the host cell. The “simplest” of positive-strand RNA virus genomes are translated as single polyproteins that are proteolytically cleaved into individual functional protein subunits by virus-encoded proteases and, in certain instances (e.g., flaviviruses), host proteases. Some of these viruses have well-defined proteolytic intermediates with differential functions of the individual protein components (e.g., picornaviruses). Thus, viruses have evolved to encode complex mechanisms within a minimal genome that dictate the efficacy of polyprotein processing in order to efficiently perform the colossal number of tasks required for replication. While these intermediates are defined for picornaviruses, the study by Płaszczyca et al. (1) identified a functional precursor in orthoflaviviruses. This study provokes investigation into the presence of other functional orthoflavivirus polyprotein intermediates and the potential importance of proteostasis in other poorly investigated positive-strand RNA viruses. It also raises questions regarding the proteolytic program of polyprotein processing. What regulates the differential cleavage that allows for the production of both specific intermediates and individual protein products?

In 2018, Ding et al. expanded on previous studies that identified the ER-localized stimulator of interferon genes (STING) host protein as a restriction factor for orthoflavivirus replication (2–4). In this publication, they identified that STING is cleaved during Zika virus infection to subvert the antiviral activity. This was further confirmed to be mediated by multiple orthoflavivirus protease complexes, which highlighted several intriguing results. First, this study showed that orthoflaviviral proteolytic cleavage of STING occurs at a non-consensus cleavage site (Y-R|G), rather than the dibasic—glycine/serine consensus motif present throughout the viral polyprotein. To my knowledge, this was the first example of the intracellular substrate flexibility of orthoflavivirus proteases. Thus, the study by Ding et al. indicated that orthoflavivirus proteases can cleave non-consensus sequences in antiviral host proteins during infection. What other host proteins are targeted for cleavage to promote infection? What is the degree of inherent substrate flexibility of orthoflavivirus proteases during infection? Interestingly, this study also showed that the yellow fever virus protease, which targets the same consensus motif in the polyprotein, does not cleave STING upon exogenous expression. Thus, there must be additional orthoflavivirus-specific determinants for protease cleavage that have yet to be defined.

Collectively, these studies have influenced a major focus of my research on viral proteases encoded by orthoflaviviruses, enteroviruses, and astroviruses. I am fortunate to have built a career in academic science with the opportunity to train the next generation of energetic scientists, which will be among the most resilient and perseverant scientists in modern history. Thus, it is imperative that we continue to support and encourage their progress by contributing to innovative science that can inspire the foundations for their independent careers—hopefully to be discussed in an mSphere of Influence article during the early years of their careers.
